# Clinical and Genetic Features of Chinese Adult Patients With Chronic Non-Bacterial Osteomyelitis: A Single Center Report

**DOI:** 10.3389/fimmu.2022.860646

**Published:** 2022-03-29

**Authors:** Mengzhu Zhao, Di Wu, Keyi Yu, Min Shen

**Affiliations:** ^1^ Department of Rheumatology, State Key Laboratory of Complex Severe and Rare Diseases, Peking Union Medical College Hospital, Chinese Academy of Medical Sciences & Peking Union Medical College, National Clinical Research Center for Dermatologic and Immunologic Diseases (NCRC-DID), Key Laboratory of Rheumatology and Clinical Immunology, Ministry of Education, Beijing, China; ^2^ Department of Orthopedic Surgery, State Key Laboratory of Complex Severe and Rare Diseases, Peking Union Medical College Hospital, Chinese Academy of Medical Sciences & Peking Union Medical College, Beijing, China

**Keywords:** chronic non-bacterial osteomyelitis (CNO), chronic recurrent multifocal osteomyelitis (CRMO), autoinflammatory disease, TNF-α inhibitor treatment, gene variant

## Abstract

**Objectives:**

Chronic non-bacterial osteomyelitis (CNO) is a rare polygenic autoinflammatory bone disease. We aimed to characterize the clinical manifestations and gene variants of Chinese adult patients with CNO.

**Methods:**

By reviewing data of all CNO patients being diagnosed and followed up at the Center for Adult Autoinflammation Diseases, Department of Rheumatology, Peking Union Medical College Hospital, clinical and genetic features of these patients were evaluated and concluded.

**Results:**

The median age of disease onset was 19 (6-64) years old, and adult-onset was observed in 6 (60%) patients. The mean time of diagnosis delay was 92 ± 78 months. The common symptoms were bone pain (10, 100%), fever (9, 90%), and arthritis (6, 60%). In total, there were 54 skeletal lesions, and each patient had no less than 2 lesions. The most frequently affected sites included lower limbs (20.5%), mandible, vertebrae and pelvis (17.5%, separately). Variants of 4 genes were detected in our study including *COL1A1*, *PSTPIP1*, *LRP5* and *CLCN7*. In seven patients who were treated with combination therapy containing tumor necrosis factor (TNF) α inhibitors, five (55.6%) had a complete response and 2 (44.4%) had a partial response.

**Conclusion:**

This is the first and largest case series of CNO in the Chinese adult patients. Four novel genetic mutations potentially associated with CNO were identified. Notably, CNO should be considered in the differential diagnosis of adult patients with long disease course and recurrent multifocal osteomyelitis of unknown cause, and these patients might benefit from combination therapy containing TNFα inhibitors.

## Introduction

Chronic non-bacterial osteomyelitis (CNO) (formerly called chronic recurrent multifocal osteomyelitis, CRMO), is a rare polygenic and multifactorial autoinflammatory bone disease, which belongs to a new branch of autoinflammatory diseases, with a morbidity rate of 1/10000 (1–4). CNO is usually a pediatric disease with an average disease-onset age of 10 to 12 years old, and is seen more frequently in girls (4–6). The most common clinical manifestation is recurrent episodes of pain in multiple bones throughout the body. Although the positions of skeletal involvement vary according to the literature, the lower limbs are the most frequently involved sites (4, 6, 7). Multiple organs and tissues are also affected, including inflammatory bowel disease, acne vulgaris, psoriasis, palmoplantar pustulosis and pyoderma gangrenosum (6, 8–12), accompanied by systemic symptoms such as fever and fatigue (8, 13). Laboratory tests are lack of specificity, and there have been no definite gene mutations associated with CNO (14, 15). The prognosis is usually good, but disability and deformity may occur in severe cases (8, 14). Adult-onset patients were occasionally described (16–18). However, cases of adult-onset or diagnosis during adulthood have been seldom reported in the Chinese population in English literature. Herein, we aimed to characterize the clinical and genetic features of Chinese adult patients with CNO.

## Patients and Methods

### Patients

From April 2015 to February 2021, ten adult (≥16 years) patients were diagnosed as CNO at the Department of Rheumatology, Peking Union Medical College Hospital.

The diagnostic criteria for CNO (5) are: (a) repeated multiple bone pain or radiographic evidence of multiple bone destruction with osteosclerotic changes throughout the body; (b) disease duration >6 weeks; (c) exclusion of other diseases such as tumors, infections, immunodeficiency diseases, and monogenic autoinflammatory diseases. Synovitis, acne, pustulosis, hyperostosis and osteitis (SAPHO) syndrome were excluded in this study.

This research was approved by the Institutional Review Board of Peking Union Medical College Hospital and performed according to the Declaration of Helsinki. Informed consents have been obtained from all participants.

### Methods

Demographic information and complete clinical data were documented at the time of diagnosis. Whole exome sequencing using next-generation sequencing (Joy Orient Translational Medicine Research Centre Co., Ltd, Beijing, China) was performed in all patients. The changes were recorded before and after treatment, including 36-item short-form (SF-36), physician’s global assessment (PGA), visual analogue scale (VAS) and inflammatory markers.

Complete response (CR) was defined as 80% or more remission of clinical symptoms and normalization of inflammatory markers after treatment. Partial response (PR) was defined as 20%-80% remission of clinical symptoms after treatment. Both CR and PR were considered effective.

## Results

### Clinical and Genetic Features of Adult Patients With CNO in Our Cohort

#### Baseline Demographic Data

**Figure 1:**
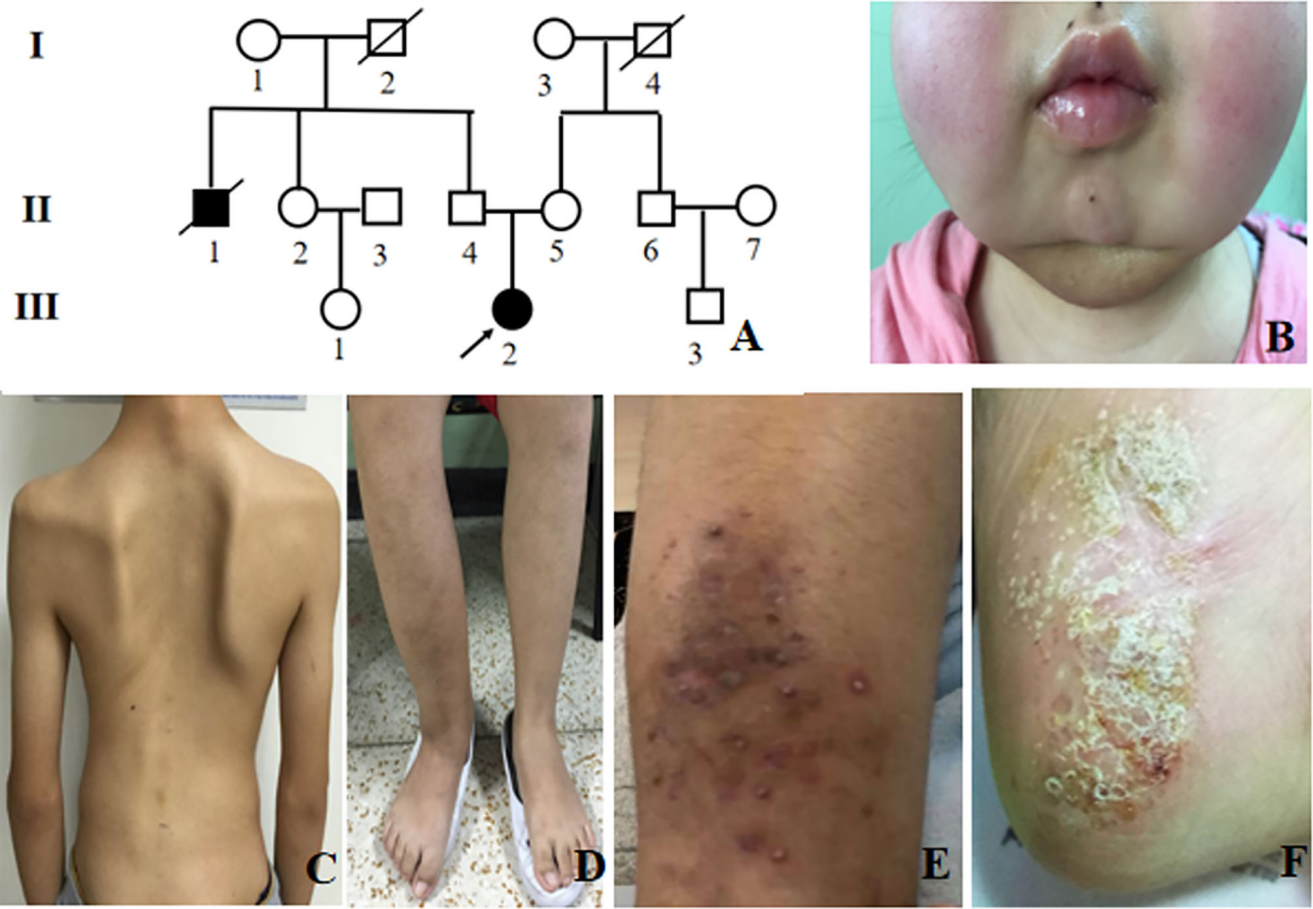
Clinical presentation of CNO patients. **(A)** Pedigree of *patient 6*. The a*rrow* indicates the proband. *Black symbols* indicate affected individuals; **(B)** Bilateral mandible involvement; **(C)** Spinal deformity; **(D)** Right tibiofibular deformity; **(E)** Psoriatic rash; **(F)** Plantar pustules.

Among 10 adult CNO patients, the gender ratio of male to female was 1: 1. The median age of disease onset was 19 (6-64) years old, and adult-onset was observed in 6 patients (60%). The median age of disease diagnosis was 22 (13-68) years old. The mean time of diagnosis delay was 92 ± 78 months. These 10 adult CNO patients were all Chinese, including 8 Chinese Han nationality, and one each Manchu and Mongolian nationality (**Table 1**). There was only one patient who had a family history of CNO (**Figure 1A**).

**Table 1:** Baseline demographic data and clinical features of Chinese adult patients with CNO.

Patient	1	2	3	4	5	6	7	8	9	10
Gender	M	F	M	F	F	F	M	M	F	M
Nationality	Han	Han	Han	Han	Han	Han	Mongolian	Han	Manchu	Han
Age at onset (years old)	23	18	20	31	64	6	9	20	6	11
Diagnosis delay (months)	8	18	137	242	16	144	48	156	116	36
Family history	–	–	–	–	–	+	–	–	–	–
Fever	+	+	+	+	+	+	+	+	–	+
Palmoplantar pustulosis	–	–	–	+	–	+	–	–	–	–
Psoriasis	–	–	–	–	–	–	–	–	+	–
Arthritis	+	+	+	–	+	–	+	+	–	–
Bone pain	+	+	+	+	+	+	+	+	+	+
Elevated ESR/CRP	+	+	+	+	+	+	+	+	+	+
Treatments										
NSAIDs	+	+	+	+	+	–	+	–	+	–
Steroids	–	+	+	–	–	+	–	–	–	–
DMARDs	–	–	+	+	+	+	–	+	+	+
Bisphosphonate	+	–	+	+	+	+	–	+	+	+
TNFα inhibitors	–	–	+	+	+	+	–	+	+	+
Efficacy	PR	PR	CR	CR	CR	CR	PR	PR	CR	PR

NSAIDs, nonsteroid anti-inflammatory drugs; DMARDs, Disease modifying anti-rheumatic drugs; PR, partial response, symptom relief of 20% to 80%; CR, complete response, 80% or more relief of symptoms. ESR, erythrocyte sedimentation rate; CRP, C-reactive protein. DMRADs, Disease modifying anti-rheumatic drugs; TNF, Tumor necrosis factor.

#### Clinical Manifestations

The most frequent symptoms of these patients were bone pain (10, 100%) and low to moderate fever (9, 90%), followed by arthritis (6, 60%). Skeletal deformities were present in 2 patients (20%) (**Figures 1B-D**). Totally, 5 patients had mucocutaneous disorders, of which two had both rash and stomatitis, two each had skin lesions, and one had mucous membrane involvement alone. Skin lesions manifested as palmoplantar pustulosis, psoriasis (**Figures 1E, F**), eczema, flaky nail shedding and perionychium desquamation. Other clinical manifestations included fatigue, weight loss, sweating, and conjunctivitis. All patients had more than one site of skeletal lesion, and 8 (80%) had bilateral lesions. A total of 54 skeletal sites were revealed in these ten patients (**Table 2**), among which lower limbs were the most common with an incidence of 20.5%. The remaining common sites were mandible, vertebrae and pelvis (17.5%, separately), followed by ribs (13%), skull (5.6%), radius (5.6%), zygoma (3.7%), and clavicle (3.7%). Nasal bone, humerus, and scaphoid (1.9%, respectively) were relatively less affected.

**Table 2:** Characteristics of bone involvements and gene variants of Chinese adult patients with CNO.

Patient	1	2	3	4	5	6	7	8	9	10
Involved sites										
Skull	–	+^#^	–	–	–	–	–	+	–	–
Nasal bone	–	+	–	–	–	–	–	–	–	–
Zygoma	–	–	–	–	–	+^#^	–	–	–	–
mandible	+^#^	–	–	–	–	+^#^	–	+^#^	+^#^	–
Clavicle	+	–	–	–	–	+	–	–	–	–
Ribs	–	+	–	+^ω^	+	–	–	–	–	–
Radius	+	–	–	–	–	+^#^	–	–	–	–
Humerus	–	–	–	–	–	–	–	–	–	+
Vertebrae	–	–	–	+^#^	+^ω^	–	+	–	–	–
Pelvis	–	+^#^	–	+	+	–	+^§^	–	–	–
Femur	+	+	+^#^	–	–	–	+	–	–	+
Tibia	+^#^	–	+	–	–	–	–	–	+	–
Fibula	–	–	–	–	–	–	–	–	+	–
Scaphoid	–	–	–	–	–	–	–	–	+	–
Gene variants (Heterozygous)	–	*PSTPIP1* c.773G>C	*PSTPIP1* c.*121C>T	*CLCN7* c.*228C>T	–	*COL1A1* (P739A)	*PSTPIP1* (c.*121C>T)	–	*LRP5* (G1055R, R1414H)	–
MAF		0.015	<0.0009	<0.016		<0.00052	<0.0009		0.000024	

^#^Bilateral or two different skeletons involved; ^§^Four different ribs skeleton involved; ^ω^Five different ribs skeleton involved; MAF, minor allele frequency. *Position after the translation stop.

All of the ten patients underwent computed tomography (CT) scan, and 9 had abnormal findings, including bone destruction (7, 70%), and marginal osteosclerosis (7, 70%). Bone marrow edema which suggested osteomyelitis was shown in all of the 8 patients who underwent magnetic resonance imaging (MRI). Among these patients, there was one patient who had no abnormality on CT, while osteomyelitis was shown by MRI. Bone scintigraphy was performed in 9 patients, and abnormal radioactive concentrations at the lesion sites were seen with a 100% positive rate. All of the 10 patients underwent bone biopsy, which indicated osteomyelitis (6, 60%), fibrotic changes (6, 60%), and osteonecrosis (2, 20%). All bacterial culture of bone tissue was negative. No patients met the diagnostic criteria of tumors, infections, autoimmune diseases or any other autoinflammatory diseases, based on bone biopsy, bacterial culture of bone tissue and other necessary examinations.

Proline-serine-threonine phosphatase-interacting protein 1 (*PSTPIP1)* gene variants were found in three patients, and chloride channel 7 (C*LCN7*), collagen type I alpha 1 (*COL1A1)*, and low-density lipoprotein receptor-related protein 5 (*LRP5)* gene variants were identified each in one patient (**Table 2**). All the gene variants were rare, with the minor allele frequency (MAF) less than 0.02 according to database of SNP (https://www.ncbi.nlm.nih.gov/snp/), 1000 Genomes Project (China), and E_X_AC (https://ngdc.cncb.ac.cn/databasecommons/database/id/3774). *PSTPIP1* gene variants included two with a C/T transition in exon 15 and one with G/C substitution in exon 11, with MAF <0.05 in the Asian population. *CLCN7* gene c.*228C>T (exon 25, rs143364973) was found in patient 4, with the MAF less than 0.016. *COL1A1* gene P793A (exon 32) variant was detected in patient 6, which is characterized by a transition from C to G, resulting in proline taking the place of alanine at residue 739. In addition, two novel variants of *LRP5* in patient 9 were found, each form asymptomatic parents. One is G1055R (c.3163G>C) in exon 14, and the other is R1414H (c.4241G>A) in exon 20. By using silico analysis algorithm, variants in *PSTPIP1* and *CLCN7* were predicted to be uncertain significance, while variants in *COL1A1* and *LRP5* were damaging and pathogenic.

#### Treatment and Outcome

As for the treatment, only one patient was once given non-steroid anti-inflammatory drugs (NSAIDs) alone. Nine patients were treated with a combination therapy of disease modifying anti-rheumatic drugs (DMARDs), bisphosphonates, NSAIDs. Seven patients were also given tumor necrosis factor (TNF) α inhibitors (**Table 1**).

**Figure 2:**
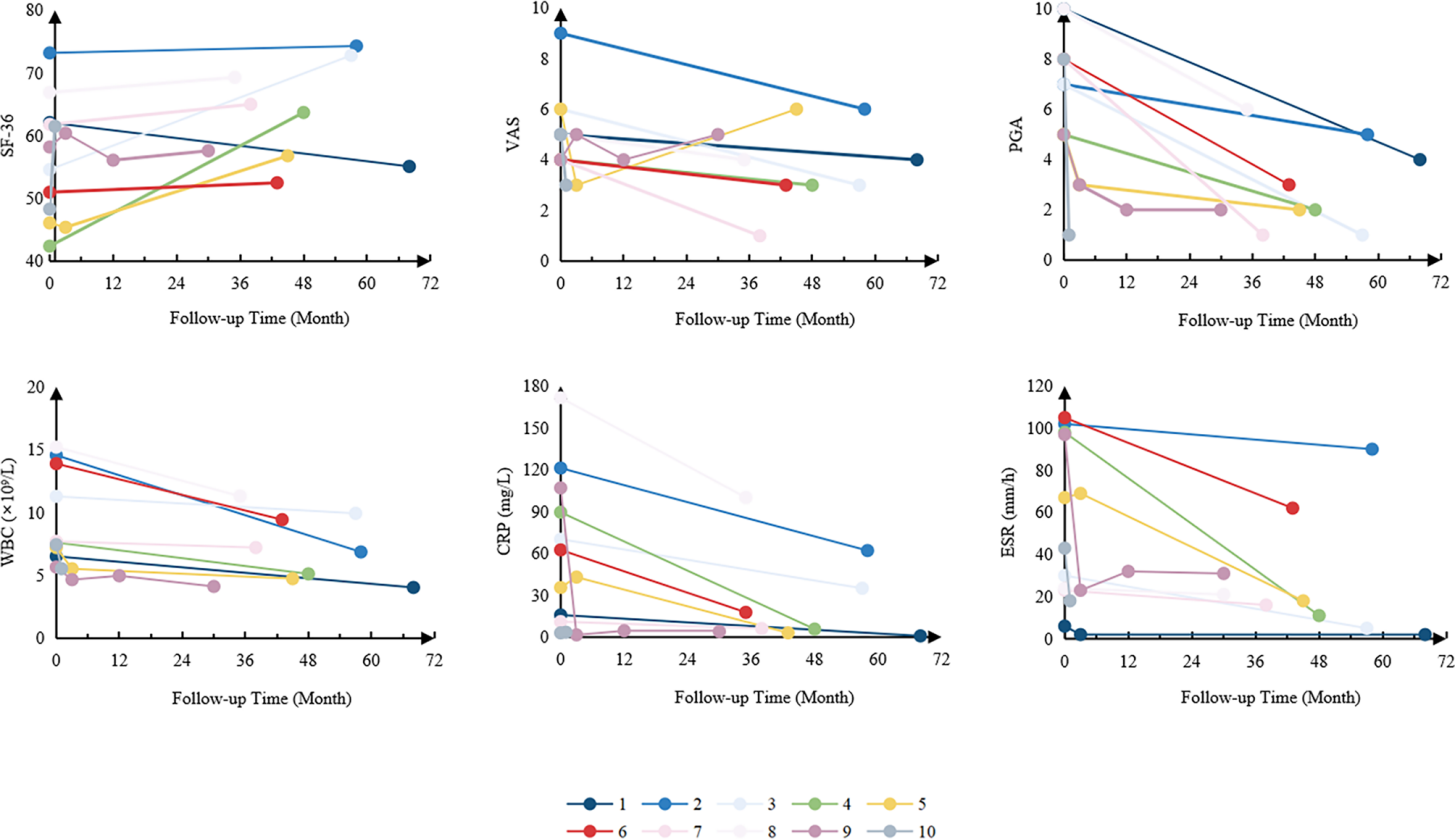
Changes in disease activity of the patients. SF-36, 36-item Short Form; VAS, visual analogue scale; PGA, physician global assessment; WBC, white blood cells; CRP, C-reactive protein; ESR, erythrocyte sedimentation rate.

Among the patients with timely follow-up, the mean follow-up time was 42 ± 19 months. The average levels of acute-phase reactants, the scores of VAS, PGA and SF-36 were collected to measure the disease activity. Overall, white blood cells (WBC), C-reactive protein (CRP), erythrocyte sedimentation rate (ESR), the scores of VAS, PGA and SF-36 significantly improved after treatments (**Figure 2**). It is worth mentioning that among the patients using TNFα inhibitors, five (55.6%) had got CR, and 2 (44.4%) PR.

## Discussion

CNO usually begins in childhood, but adult-onset patients have also been reported (16–21). Two-thirds of the patients in our study were adult-onset, which might be related to the fact that our center mainly admitted adult patients. In our study, the mean time from onset to diagnosis was 92 ± 78 months, with the longest 242 months. Some patients even had bone deformation and osteolysis due to delayed diagnosis and improper treatments. Thus, we suggest that early recognition of CNO and detailed differential diagnosis is critical in patients with unknown reason of bone pain and fever to avoid irreversible damage.

It was reported that adult CNO patients were more likely to have mucocutaneous involvement, solely lytic bone lesion and worse response to NSAIDs and glucocorticoids than pediatric patients (5). Consistent with the published literature, we found that patients in our study tended to have more mucocutaneous involvement (50% *vs*. less than 12%) and had poorer response to NSAIDs (57% *vs*.73%) compared with the French center (mainly pediatrics) (5). However, all of our patients had no less than 2 bone lesions, which might be related to the long disease course. It is worth noting that CNO is an exclusionary diagnosis and is important to distinguish from infections and tumors (2). We suggest that bone biopsy and thorough etiological examination (smear and culture) are very necessary for the differential diagnosis (22).

Although it has been indicated that SAPHO syndrome and CNO are the same diseases occurring at different ages, but this theory has not been sufficiently confirmed (4, 15, 16, 18, 23, 24). It is generally believed that SAPHO syndrome starts in adults and clinically manifests as osteomyelitis which is similar to CNO (25). However, in terms of pathogenesis, CNO is mainly associated with increased activation of innate immune mechanisms and responds to blockade of these mechanisms, but patients with SAPHO respond to blockade of the T lymphocyte-derived cytokine IL-17A, which suggests that adaptive immunity plays a greater role in SAPHO, compared to CNO (15). Secondly, regarding skeletal involvement and imaging presentation, SAPHO mainly affects patients’ sternum, sternoclavicular joints, sacroiliac joints, accompanied by various skin lesions, while CNO mainly involves the metaphyses of long bones (25). For imagings, SAPHO is characterized by hyperostosis and osteitis, while CNO features osteolytic lesions (25, 26). Besides, skin manifestations are major features of SAPHO, and considering CNO without skin lesions as SAPHO would confuse clinicians with correct diagnosis (16). Taken together, CNO in adults should be distinguished diagnostically from SAPHO syndrome (16). In this study, no adult-onset patients had skin involvement, and their clinical manifestations were consistent with the diagnosis criteria of CNO proposed in 2007 (27). Therefore, we thought that CNO, rather than SAPHO syndrome, should be diagnosed in these patients.

No clearly associated gene mutations have been found in CNO patients to date. The *PSTPIP1* gene, located on chromosome 15, encodes a protein whose impaired phosphorylation causes enhanced inflammasome activation and increased release of IL-1, IL-18 and other proinflammatory cytokines (28). It has been proved that *PSTPIP1* is the pathogenetic gene for pyogenic arthritis, pyoderma gangrenosum, and acne (PAPA) syndrome, an autosomal dominantly inherited monogenic autoinflammatory disease (28, 29). However, none of the three patients in our study were consistent with the clinical features of PAPA syndrome. It has been proved that PSTPIP1 regulates the podosome and sealing zone disassembly in osteoclasts through PSTPIP1/PTPN6/SHIP1/2 complex upon Src-dependent phosphorylation and PI (3–5)P3 signaling which plays an important role in bone remodeling (30). Therefore, even these variants were predicted to be uncertain significance, we still recommend further functional studies to clarify the pathogenic role of them.

*CLCN7* gene is located on chromosome 16p13.3 and is usually associated with type II autosomal dominant osteoporosis (ADO2), which features adult-onset and thickening of the vertebral endplates and endopelvic bone (31, 32). The mentioned variant for osteopetrosis remains uncertain by Illumina Clinical Services Laboratory Variant Classification Criteria 13, but there are no related case reports of the gene, cDNA changes, or amino acid changes according to Clinvar database (https://www.ncbi.nlm.nih.gov/clinvar/). Although patient 4 had an adult onset of disease, there were no clinical manifestations associated with AOD2, so the patient could not be diagnosed as AOD2 clinically. Further efforts may be required to verify the mutation in clinical significance.

The *COL1A1* gene locates on chromosome 17q21.31-q22, and 252 variants have been reported in the Clinvar database. Most of the variants are related to osteogenesis imperfecta (OI), and the rest may be linked to Caffey disease and Ehlers-Danlos syndrome (EDS) (33). Also, it has been indicated that *COL1A1* may be related to developmental dysplaisa of the hips (DDH) (34). In fact, the *COL1A1* P793A variant occurs within the major ligand-binding region (MLBR) 2, which includes sites important for collagen assembly, cleavage, and binding, and can further affect the bone strength and flexibility. Moreover, it has been proved that glycine substitution in MLBR is harmful (35). Specially, the similarities of Caffey disease and CNO, such as a recurrent, remitting course, bone lesions at the same sites, inflammatory changes in bone and adjacent soft tissues, and tubular bones involvement, indicate that both of them may share the same downstream signaling pathway (36). Since the MAF of P793A in patient 6 was less than 0.00052 in the Asian population, and it is predicted to be probably damaging when using a silico analysis algorithm, we think the variant might play a part in the pathogenesis of CNO.

Interestingly, by using Clustered Regularly Interspaced Short Palindromic Repeats- CRISPR-associated protein 9 (CRISPR-Cas9) to knock out the *LRP5* gene, the LRP5-/-mice showed low bone mass, decreased bone mineral density, and decreased bone size (37). Except for the evidence mentioned above, a lot of increasing studies showed that LRP5, a co-receptor of the Wnt pathway, played a prominent role in bone metabolism and remodeling, which was related to the osteoporosis, osteonecrosis of the femoral head, steroid-induced osteonecrosis of the femoral head, osteoporosis-pseudoglioma syndrome and other possible disease (38–43). Heterozygous deletion of the *LRP5* gene in mice presented with alters profile of immune cells (44). Moreover, it has been proved that Wnt signaling pathway contributes to bone erosion and cartilage degradation in rheumatoid arthritis (45, 46). Besides, both of the variants in patient 9 were with a MAF of <0.0009 in the Asian population, and their function were damaging according to predictions by the silico analysis algorithm. Overall, the variants of *LRP5* gene might also have a certain effect in CNO.

## Conclusion

This is the first and largest case series of CNO in the Chinese adult patients. CNO should be suspected in patients with unexplained bone pain. Pathological and etiological examination through bone biopsy is critically helpful for the differential diagnosis of CNO from infections and tumors. Four novel gene mutations related to CNO were found in the cohort, and further research is needed to identify the relationship between genes and phenotypes. A combination therapy containing TNFα inhibitors might be effective in the treatment of CNO.

## Data Availability Statement

According to national legislation/guidelines, specifically the Administrative Regulations of the People’s Republic of China on Human Genetic Resources (http://www.gov.cn/zhengce/content/2019-06/10/content_5398829.htm, http://english.www.gov.cn/policies/latest_releases/2019/06/10/content_281476708945462.htm), no additional raw data is available at this time. Data of this project can be accessed after an approval application to the China National Genebank (CNGB, https://db.cngb.org/cnsa/). Please refer to https://db.cngb.org/, or email: CNGBdb@cngb.org for detailed application guidance. The accession code PRJCA008110 should be included in the application.

## Ethics Statement

The studies involving human participants were reviewed and approved by Ethics Committee of PUMCH. The patients/participants provided their written informed consent to participate in this study.

## Author Contributions

MZ, KY, and MS contributed to conception and design of the study. DW, KY, and MS organized the database. MZ performed the statistical analysis. MZ wrote the first draft of the manuscript. All authors contributed to manuscript revision, read, and approved the submitted version.

## Funding

This work was supported by the Natural Science Foundation of Beijing (Grant No.7192170); the Chinese Academy of Medical Sciences Innovation Fund for Medical Sciences (CIFMS) (Grant No. 2017-I2M-3-001); the Peking Union Medical College Hospital Foundation for Distinguished Young Scholars (Grant No. JQ201705); and the National Key Research and Development Program of China (Grant No.2016YFC0901500; 2016YFC0901501).

## Conflict of Interest

The authors declare that the research was conducted in the absence of any commercial or financial relationships that could be construed as a potential conflict of interest.

## Publisher’s Note

All claims expressed in this article are solely those of the authors and do not necessarily represent those of their affiliated organizations, or those of the publisher, the editors and the reviewers. Any product that may be evaluated in this article, or claim that may be made by its manufacturer, is not guaranteed or endorsed by the publisher.
